# Spatial high-resolution socio-energetic data for municipal energy system analyses

**DOI:** 10.1038/s41597-019-0233-0

**Published:** 2019-10-30

**Authors:** Jann M. Weinand, Russell McKenna, Kai Mainzer

**Affiliations:** 1 Chair of Energy Economics, Karlsruhe Institute of Technology, Karlsruhe, Germany; 2 DTU Management, Technical University of Denmark, Lyngby, Denmark

**Keywords:** Energy economics, Energy modelling, Photovoltaics, Geothermal energy, Wind energy

## Abstract

In the context of the energy transition, municipalities are increasingly attempting to exploit renewable energies. Socio-energetic data are required as input for municipal energy system analyses. This Data Descriptor provides a compilation of 40 indicators for all 11,131 German municipalities. In addition to census data such as population density, mobility data such as the number of vehicles and data on the potential of renewables such as wind energy are included. Most of the data set also contains public data, the allocation of which to municipalities was an extensive task. The data set can support in addressing a wide range of energy-related research challenges. A municipality typology has already been developed with the data, and the resulting municipality grouping is also included in the data set.

## Abstract (summary)

**Table:** 

Measurement(s)	Demographic Data • vehicle • renewable energy
Technology Type(s)	digital curation
Sample Characteristic - Environment	city
Sample Characteristic - Location	Germany

Machine-accessible metadata file describing the reported data: 10.6084/m9.figshare.9933890

## Background & Summary

National targets in energy policy are leading to a radical change in the energy sector. The associated expansion of renewable energies is mainly decentralised, which also applies to the owners and operators of energy plants: private individuals increasingly invest in renewable energy systems or form so-called citizen-energy cooperatives^1^. More and more municipalities are striving to exploit renewable decentralised energy generation. Thereby they participate in municipal projects like “Bioenergy villages” and “100%-Renewable-Energy-Communities”^2^.

In the course of the growing interest in renewable energy systems, an increasing number of energy system analyses for the development of climate protection plans are conducted at the municipal level. However, many municipalities lack both the financial resources and the expertise to determine the potential for renewables or develop effective climate protection plans^3^. These municipalities would benefit from studies on their suitability for decentralised energy systems.

In addition, a growing number of energy system models are used whose input values are based on public data^4,5^. The availability of data can therefore also support the development of energy system models.

We recently grouped the 11,131 German municipalities with regard to their suitability for decentralised energy^6^. The cluster analysis included 38 socio-energetic indicators which comprised data on the energy consumption sectors “Private Households” and “Transport” as well as data to estimate the potential for renewable energies. Among the data on renewable energies are spatial high-resolution photovoltaic^7^, wind^8^ and hydrothermal^6^ potentials. The hereby-published dataset contains all indicators and the resulting cluster composition for all German municipalities.

The dataset^9^ enables energy researchers to conduct studies at municipal and national levels without having to obtain and synthesize a large amount of data. For example, the cluster composition can help to transfer results from energy system analyses of individual municipalities to other, similar municipalities.

## Methods

In the following, the various methods for determining and allocating the data to the municipalities are described. A distinction is made between data on census, mobility and renewable energies as well as cluster data (cf. Online-only Table 1).

### Census data

**Fig. 1:**
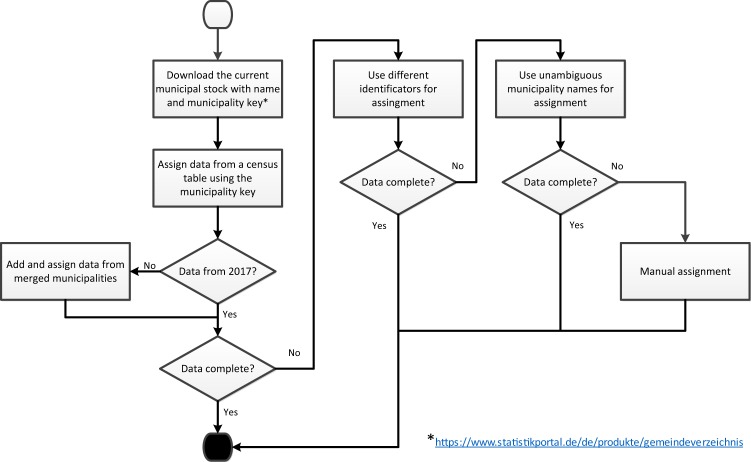
Procedure for consolidating the census data.

Despite the fact that the census data are public, consolidating the data is an extensive task^6^. This is related on the one hand to different identifiers for the municipalities in the various census data tables. On the other hand, the number of municipalities in Germany is constantly reducing as several municipalities are merged into one. In addition, the data tables are rarely complete and data are missing for individual municipalities. Figure 1 shows the procedure for consolidating the data for one census table. The data were assigned to the municipalities from the municipal register of 2017 (https://www.statistikportal.de/de/produkte/gemeindeverzeichnis). Some census data tables, however, do not exist for 2017. Apart from the table from 2011, which was needed for the population development, the oldest table used is from 2014. In these cases, municipalities are indicated which no longer existed in 2017 as they were merged into one municipality. Then the values in the older tables were combined and assigned to the newly established municipality. If not all municipalities could be assigned a value after these steps, other identification numbers than the municipality key were applied. In case this was not sufficient, the names of the municipalities were used to assign the data. However, there are many municipalities with the same name in Germany. Therefore, attention was paid to the unambiguity of the names, e.g. by combining them with numbers from the municipality key. In a few cases, data could still not be assigned after these steps (for less than 2% of municipalities). Then the data was manually collected, e.g. via web searches.

The population density in the data set is related to the municipal area. Population density in relation to settlement areas would be an even more relevant indicator for energy system analyses as this data could be used to estimate costs for district heating networks. This more accurate data is published in a parallel study^10^.

### Mobility data

The mobility data in the municipalities in Germany are published in PDF format^11^. The delivery of the data in a processable format such as csv is subject to a fee. Therefore, the data was transferred from the PDF to a csv chart. Subsequently, the data could be allocated according to the same procedure as for the census data (cf. Fig. 1).

### Renewable energy data

In earlier studies, potentials for electricity generation from photovoltaics, wind and geothermal energy were determined. The methods are explained in more detail below.

#### Technical photovoltaic potential

**Fig. 2:**
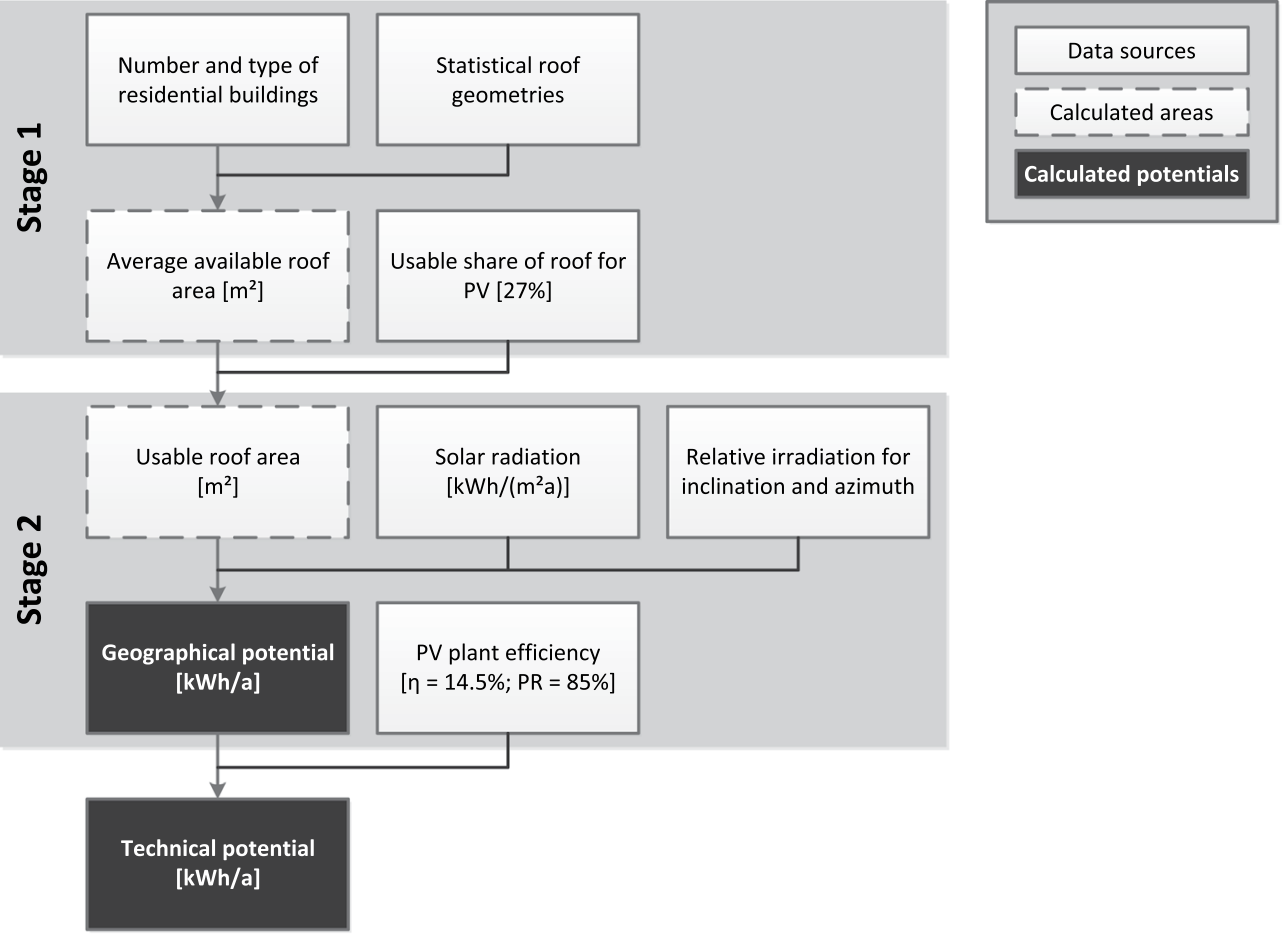
Methodology for the assessment of technical PV potentials. References are used for the number and type of residential buildings^20^, the statistical roof geometries^21^, the solar radiation^22^ and the relative irradiation for inclination and azimuth^23^.

A high-resolution determination of the technical potential of residential roof-mounted photovoltaic systems for each municipality in Germany was presented by Mainzer *et al*.^7^. The method for calculating these potentials consists of two stages (see Fig. 2): first, the usable roof area in each municipality is calculated using statistical data such as the number and type of residential buildings as well as statistical data on roof geometries and the usable share of the roofs for photovoltaic systems. Next, the calculated roof area together with assumptions on the distribution of inclination and azimuth angles is combined with each municipalities’ solar radiation data as well as the relative irradiation for specific inclination and azimuth angles to calculate the geographical potential. Combined with the technical PV plant efficiency, the technical potential for each municipality can then be inferred. Since the calculation method is described in detail in Mainzer *et al*.^7^, all assumptions can easily be adjusted to the readers own preferences.

Non-residential buildings could be considered with our newer, more detailed methods^12^, which, however, could not yet be applied to the whole of Germany due to the higher resource demand (calculation time and storage capacity) of these methods.

#### Technical wind potential

**Fig. 3:**
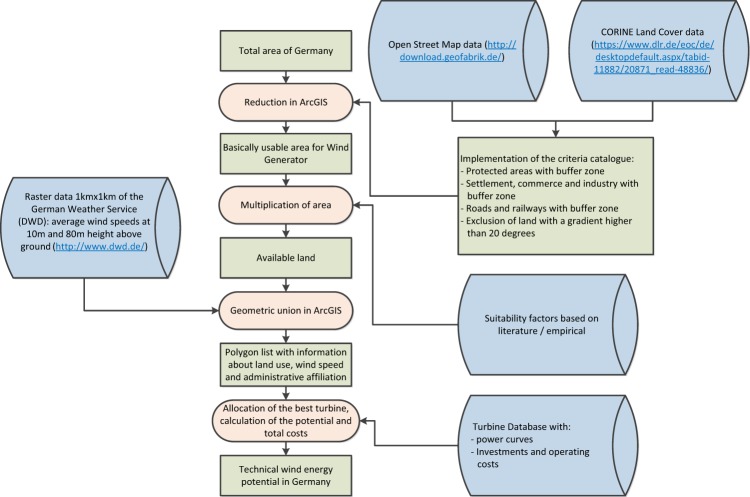
Methodology for the assessment of technical wind energy potentials.

In another study, the potentials for onshore wind in Germany were calculated^8^. The applied method employs multiple data sources for land use categories, annual average wind speeds and techno-economic wind turbine data for several hundred plants as shown in Fig. 3. By excluding unsuitable areas, such as areas with a gradient above 20°, urban areas and natural parks, and inserting a buffer area around these as well as residential, commercial and industrial areas, the technically feasible area for onshore wind energy was determined. These remaining areas are associated with suitability factors based on empirical values and annual average wind speed data at 1 km^2^ resolution. The final step, and one key innovation of the study, involved matching a wind turbine to the polygon types (combination of wind speed and land use category) based on the lowest levelized costs of electricity (LCOE). The results represent the lowest cost realization of the technical potential for onshore wind in Germany, based on the then (2013) state of the art in turbine technology.

The technical onshore wind potential data was aggregated to postcode level in McKenna *et al*.^8^. With the help of the geo-information system QGIS, the postcode areas were intersected with the administrative municipal areas from 2017. In this way, the wind potentials could be divided among German municipalities.

#### Achievable hydrothermal temperature

**Fig. 4:**
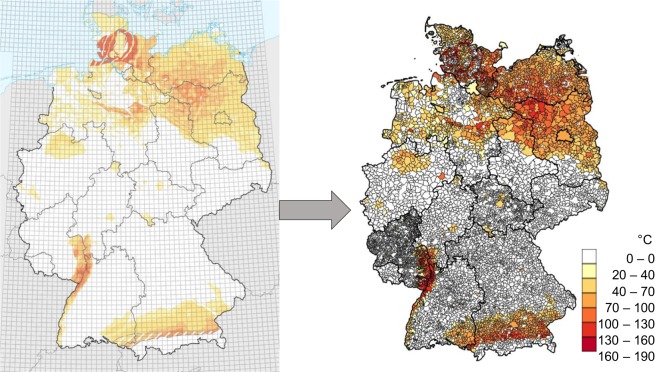
Achievable hydrothermal temperatures (°C) in Germany at depths of up to 5000 meters. Data from the GeotIS project was transferred and assigned to the German municipalities.

Figure 4 shows that the achievable hydrothermal temperatures in Germany strongly depend on the region. This means that municipalities have different hydrothermal potentials. In the GeotIS project, the hydrothermal temperatures in Germany were determined (cf. left part of Fig. 4)^13^. With the help of a raster contained in the GeotIS tool, the temperatures could be transferred manually to a spatial resolution of 8.5 km². Subsequently, achievable hydrothermal temperatures could be assigned to the municipalities. Thereby, the temperature in a municipality and closest to the centre of the municipality was assigned to the municipality. Since on the one hand the transfer of 57,535 data points from the GeotIS map was very time-consuming and on the other hand different assignment methods to the municipalities could be chosen, a data table for the hydrothermal temperatures depending on the precise coordinates is also provided^9^. These coordinates represent the intersections of the grid shown in the left part of Fig. 4.

### Cluster data

In Weinand *et al*.^6^, indicators from Online-only Table 1 were first standardised to values between zero and one to ensure that all indicators have the same weight in the cluster analysis. Subsequently, a factor analysis was used to filter out the indicators that were not relevant for the further steps. Then the municipalities were grouped using a hierarchical cluster analysis. Thereby, 26 cluster validation criteria were applied to determine an appropriate number of clusters. The ten resulting clusters can also be found in the provided data set. Among these clusters are municipality groups with a high potential for renewable energies (Cluster 3, 4, 7 and 8), with a high proportion of district heating (Cluster 1) or cities with a high population density (Cluster 2).

## Data Records

The municipality data summarised in Online-only Table 1 are available as an xls file in an online repository^9^. More than 400,000 data entries can be clearly assigned and identified on the basis of the column headings. The data for the hydrothermal temperatures are available as point coordinates in 8.5 km² resolution in a csv file^9^. The first column contains the latitudes, the first row the longitudes. The hydrothermal temperature is in the cell where latitudes and longitudes intersect. The entries are numbers from 0 to 5, where 0 means that there is no hydrothermal potential. For 1 the temperature is between 40 °C and 60 °C, for 2 between 60 °C and 100 °C, for 3 between 100 °C and 130 °C, for 4 between 130 °C and 160 °C and for 5 between 160 °C and 190 °C.

## Technical Validation

When validating the data, the heterogeneity of the “concept of municipality” as used by the different federal states becomes apparent. For example, Baden-Wuerttemberg, which accounts for about 13% of the population, has only 75 municipalities with less than 1000 inhabitants. The neighbouring Rhineland Palatinate, which accounts for 5% of the population, yet counts 1624 municipalities with less than 1000 inhabitants (https://www.statistikportal.de/de/produkte/gemeindeverzeichnis). In an international context, the “concept of municipality” is even more heterogeneous. The data set thus provides high-resolution data, but the system boundary of a municipality is not necessarily the most suitable one for energy system analyses.

### Census and mobility data

The census and mobility data are official data from German authorities and are therefore assumed to be accurate. Nevertheless, we have checked these data for anomalous values. For example, in 75 municipalities the population is zero. These municipalities are “municipality-free areas”, i.e. municipalities without inhabitants. These are usually municipalities with nature reserves or military stations (for more details please refer to Weinand *et al*.^6^).

### Technical photovoltaic potential

A validation of the technical PV potential has been performed for two intermediate results, using cadastral data from the federal state of Baden-Württemberg, which comprises about 10% of the area and 13% of the residential buildings in Germany. The validation shows that the number of residential buildings from the Baden-Württemberg cadastral building data differs by just 1.65% from the publicly available statistical data. It also shows that the assumed residential building sizes as well as the total ground floor area for Baden-Württemberg agree with the building sizes extracted from the cadastral data. It can thus be assumed that the employed assumptions as well as the statistical data used represent the German building stock adequately (see Mainzer *et al*.^7^ for more details).

### Technical wind potential

The onshore wind cost-potentials were validated with other studies in the literature. With central key assumptions of this method adjusted to reflect those in BWE^14^, the results in both cases are very similar. Our study estimates a total potential area of about 41,000 km^2^ compared to the BWE’s 46,000 km^2^. In terms of the available area for wind energy in Germany, the present work with 41,613 km^2^ also agrees well with the UBA^15^ study, which determined 49,000 km^2^. The reason for the lower results in our case lies in the more conservative assumed suitability factors and offset distances from obstacles.

The discrepancy is significantly larger in relation to the installable power and generated energy, however: UBA conclude that about 1,190 GW could be installed to generate about 2,900 TWh/a (i.e. average full load hours of about 2400), compared to 367 GW and 855 TWh/a (full load hours of 2329)^8^. Whilst some of the discrepancy can be explained by the difference in the determined areas, the majority is probably due to the significantly higher turbine densities employed by UBA. The high overall turbine density is achieved through an aggregation procedure that clusters nearby polygons together (similarly to Fueyo *et al*.^16,17^). For Baden-Württemberg, McKenna *et al*.^18^ found a technical potential of 29 to 41 GW at costs between 6 and 21 €ct/kWh. This compares well to the technical potential calculated in our study of 34 GW at average costs of 9.5 €ct/kWh. Similarly, McKenna *et al*.^19^ found a potential and land area for Germany of 707 TWh and 35,700 km^2^ respectively in a similar study at the European level. The only other recent study that considered the whole of Germany was EEA (www.eea.europa.eu/), which did not differentiate between different land use categories. This explains the very high total technical potential of 4000 TWh/a identified in Germany. The calculated fraction of this potential, which by 2020 should be competitive, was found to be 258 TWh/a, which corresponds to generation costs of about 7 ct/kWh in McKenna *et al*.^8^. A direct comparison of the generation costs with EEA is not possible, however, as they were not divulged for individual countries or states.

### Achievable hydrothermal temperature

As already described above, the data for the achievable hydrothermal temperature were transferred based on the GeotIS project results^13^. On the one hand, visual inspection of the maps in Fig. 4 shows that the data was transferred correctly. On the other hand, this has also been confirmed by a sampling check.

### Cluster data

In the cluster analysis, 26 cluster validation criteria were applied to determine the number of clusters^6^. Further validation methods came to the same results as the 26 cluster criteria.

## Data Availability

Microsoft Excel version 2010 and ArcGIS version 10.1 were used to determine the technical PV and wind potentials. QGIS version 2.14.10 was used for the transfer of wind and hydrothermal potential to the municipality level. MATLAB version 2017b and Microsoft Excel version 2016 were used to compile the census and mobility data. Microsoft Excel or another program for processing CSV or XLS files is required to process the data set provided with this data descriptor. The codes for generating the data can be made available on request.

## Online-only Table

**Online-only Table 1:** Data description and references.

Indicator	References
**Census Data**	
Population [−]	https://www.statistikportal.de/de/produkte/gemeindeverzeichnis
Municipality area [km²]	https://www.statistikportal.de/de/produkte/gemeindeverzeichnis
Population development between 2010 and 2015 [%]	https://www.statistikportal.de/de/produkte/gemeindeverzeichnis, https://www.regionalstatistik.de/genesis/online
Living space per person [m²]	https://www.statistikportal.de/de/produkte/gemeindeverzeichnis, https://www.regionalstatistik.de/genesis/online
Share of single-person households in total number of households [%]	https://www.regionalstatistik.de/genesis/online
Average household size [Number of persons per household]	https://www.statistikportal.de/de/produkte/gemeindeverzeichnis, https://www.regionalstatistik.de/genesis/online
Household density [Households per km² of municipality area]	https://www.statistikportal.de/de/produkte/gemeindeverzeichnis, https://www.regionalstatistik.de/genesis/online
Share of owner-occupied apartments in total number of apartments [%]	https://www.regionalstatistik.de/genesis/online
Income per household [k€]	https://www.regionalstatistik.de/genesis/online
Share of over 65-year-olds in total population [%]	https://www.regionalstatistik.de/genesis/online
Unemployment rate [%]	https://www.statistikportal.de/de/produkte/gemeindeverzeichnis, https://www.regionalstatistik.de/genesis/online
Share of settlement and traffic area in total area [%]	https://www.regionalstatistik.de/genesis/online
Share of buildings with heating systems based on district heating [%]	https://www.regionalstatistik.de/genesis/online
Share of buildings with heating systems not based on district heating [%]	https://www.regionalstatistik.de/genesis/online
Share of buildings without heating system [%]	https://www.regionalstatistik.de/genesis/online
Share of buildings built before 1919 in total building stock [%]	https://www.regionalstatistik.de/genesis/online
Share of buildings built between 1919 and 1949 in total building stock [%]	https://www.regionalstatistik.de/genesis/online
Share of buildings built between 1950 and 1959 in total building stock [%]	https://www.regionalstatistik.de/genesis/online
Share of buildings built between 1960 and 1969 in total building stock [%]	https://www.regionalstatistik.de/genesis/online
Share of buildings built between 1970 and 1979 in total building stock [%]	https://www.regionalstatistik.de/genesis/online
Share of buildings built between 1980 and 1989 in total building stock [%]	https://www.regionalstatistik.de/genesis/online
Share of buildings built between 1990 and 1999 in total housing stock [%]	https://www.regionalstatistik.de/genesis/online
Share of buildings built between 2000 and 2005 in total building stock [%]	https://www.regionalstatistik.de/genesis/online
Share of buildings built from 2006 onward in total building stock [%]	https://www.regionalstatistik.de/genesis/online
Share of detached houses in total building stock [%]	https://www.regionalstatistik.de/genesis/online
Share of semi-detached houses in total building stock [%]	https://www.regionalstatistik.de/genesis/online
Share of terraced houses in total building stock [%]	https://www.regionalstatistik.de/genesis/online
Share of “other building types” in total building stock [%]	https://www.regionalstatistik.de/genesis/online
Population density [Inhabitants per km² of municipality area]	https://www.statistikportal.de/de/produkte/gemeindeverzeichnis
Share of 18–64-year-olds in the total population [%]	https://www.regionalstatistik.de/genesis/online
Share of forest and agricultural land in total area [%]	https://www.regionalstatistik.de/genesis/online
**Mobility data**	
Number of motor vehicles per 1,000 inhabitants	https://www.statistikportal.de/de/produkte/gemeindeverzeichnis ^11^
Number of cars per 1,000 inhabitants	https://www.statistikportal.de/de/produkte/gemeindeverzeichnis ^11^
**Renewable energy data**	
Achievable hydrothermal temperature [°C]	^13^ and own calculation
Technical PV potential per inhabitant [kWh/y]	https://www.statistikportal.de/de/produkte/gemeindeverzeichnis ^7^
Technical PV potential per km² [MWh/y]	https://www.statistikportal.de/de/produkte/gemeindeverzeichnis ^7^
Technical wind potential per inhabitant [MWh/y]	https://www.statistikportal.de/de/produkte/gemeindeverzeichnis ^8^
Technical wind potential per km² [MWh/y]	https://www.statistikportal.de/de/produkte/gemeindeverzeichnis ^8^
**Cluster data**	
Municipality groups	^6^
